# Supplementary material for: Novel Transcriptional and DNA Methylation Abnormalities of SORT1 Gene in Non-Small Cell Lung Cancer
Source: Cancers (Basel). 2024 Jun 6;16(11):2154. doi: 10.3390/cancers16112154 (PMC11171784; doi:10.3390/cancers16112154)

Supplementary Figure S3

TCGA Lung Adenocarcinoma

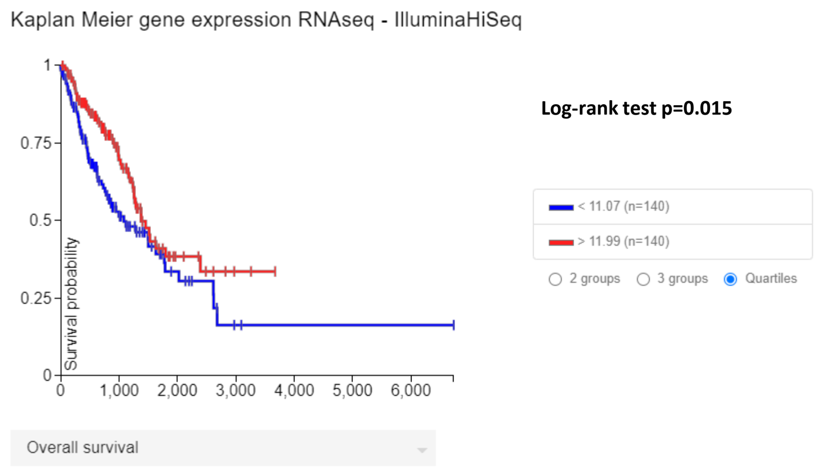

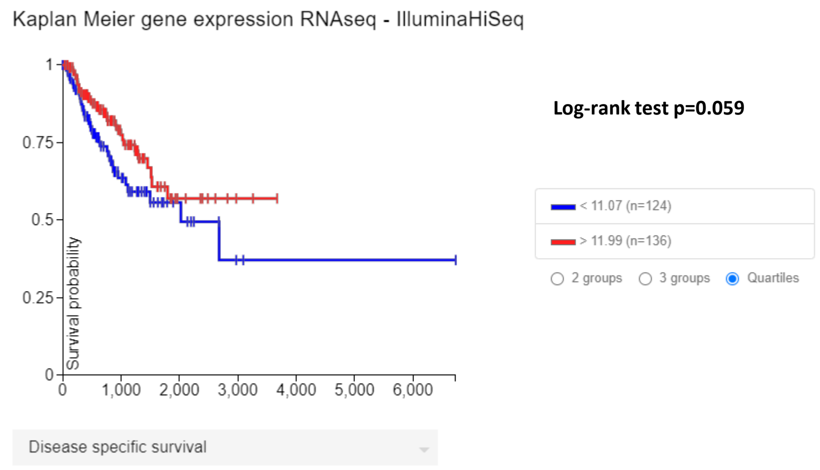

TCGA Lung Squamous Cell Carcinoma

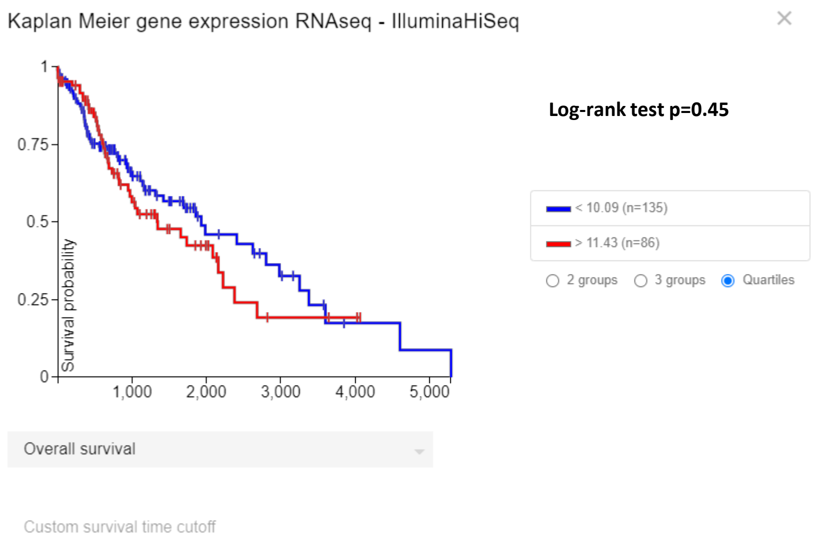

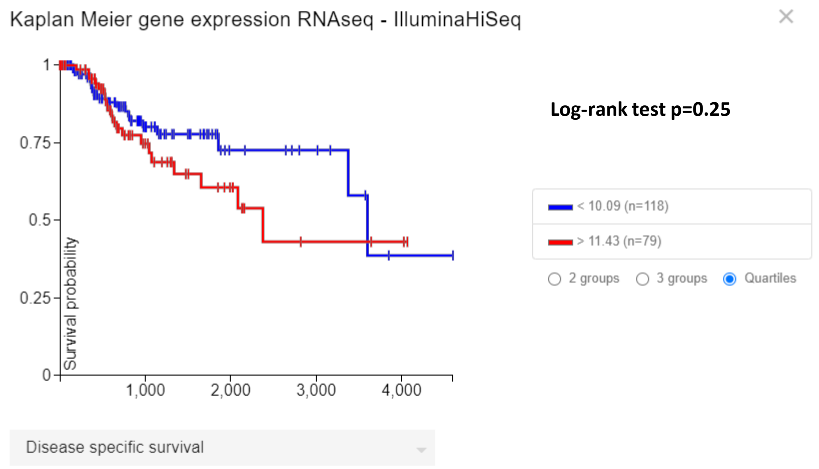

Supplement: Supplementary file 1 [file cancers-16-02154-s001.zip › Supplementary Figure S3.pdf]
