# Supplementary material for: Novel Transcriptional and DNA Methylation Abnormalities of SORT1 Gene in Non-Small Cell Lung Cancer
Source: Cancers (Basel). 2024 Jun 6;16(11):2154. doi: 10.3390/cancers16112154 (PMC11171784; doi:10.3390/cancers16112154)
Supplement: Supplementary file 1 [file cancers-16-02154-s001.zip › Supplementary Table S1.pdf]

Supplementary Table S1. Clinical characteristics of lung cancer patients in this study.

	**Expression set**	**Methylation set**
N	81	123
**Age**		
mean (s.d.)	66.59 (8.4)	65.84 (8.98)
**Gender**		
Male : Female	55:26	91:32
**Histology**		
Adenocarcinoma	25	52
Squamous	56	70
Other	-	1
**Tumour stage**		
T1	12	7
T2	57	96
T3	9	13
T4	3	2
Missing	-	5
**Nodal stage**		
N0	42	63
N1	22	35
N2	17	20
Missing	-	5
**Differentiation**		
Well	6	9
Moderate	43	76
Poor	20	34
Missing	12	4

Histology; Others is 1 NSCLC
