# Supplementary material for: Novel Transcriptional and DNA Methylation Abnormalities of SORT1 Gene in Non-Small Cell Lung Cancer
Source: Cancers (Basel). 2024 Jun 6;16(11):2154. doi: 10.3390/cancers16112154 (PMC11171784; doi:10.3390/cancers16112154)
Supplement: Supplementary file 1 [file cancers-16-02154-s001.zip › Supplementary Table S2.pdf]

Supplementary Table S2. Clinical characteristics of lung cancer patients and controls utilised for examining DNA methylation in blood.

Blood	Cases	Controls
N	254	248
**Age**		
mean (s.d.)	66.96 (7.8)	53.80 (7.05)
**Gender**		
Male : Female	145:109	140:108
**Histology**		
Adenocarcinoma	104	
Squamous Cell	113	
Small Cell	27	
Others	10	
**Tumour stage**		
T1	53	
T2	91	
T3	31	
T4	45	
Missing	34	
**Nodal stage**		
N0	96	
N1	39	
N2	49	
N3	28	
Missing	42	
**Stage**		
IA	31	
IB	39	
IIA	20	
IIB	25	
IIIA	35	
IIIB	26	
IV	43	
Missing	35	
**Differentiation**		
Well	8	
Moderate	81	
Poor	31	
Undifferentiated	1	
Missing	133	
