# Supplementary material for: Novel Transcriptional and DNA Methylation Abnormalities of SORT1 Gene in Non-Small Cell Lung Cancer
Source: Cancers (Basel). 2024 Jun 6;16(11):2154. doi: 10.3390/cancers16112154 (PMC11171784; doi:10.3390/cancers16112154)
Supplement: Supplementary file 1 [file cancers-16-02154-s001.zip › Supplementary Table S3.pdf]

Supplementary Table S3. Primer/probe sequences and amplicon size of qPCR assays used for measuring SORT1A and SORT1B expression in this study.

Locus	Primer sequence
SORT1A	Fwd: 5'-CAACAACACGCACCAGCAT-3'
	Rev: 5'-CTAGAATGACCCCAGTGCTATCTC-3'
	Probe: 5'-FAM-CAGAGGCTCAGTATCCTTGTCCTGGG-BHQ2-3'
	Product size: 86 bp
SORT1B	Fwd: 5'-TTGTTGTGAATCGCCAGACC-3'
	Rev: 5'-CAACCCAGGACAAGGATACTGA-3'
	Probe: 5'-FAM-CACCCGGAAGTCTGAAGCATGTGTTT-BHQ2-3'
	Product size: 90 bp
TBP	Fwd: 5’-GGGGAGCTGTGATGTGAAGTTT-3'
	Rev: 5'-AAACCAGGAAATAACTCTGGCTCA-3'
	Probe: 5'-TAMRA-AAGGCCTTGTGCTCACCCACCAAC-BHQ2-3'
	Product size: 96 bp
