# Supplementary material for: Novel Transcriptional and DNA Methylation Abnormalities of SORT1 Gene in Non-Small Cell Lung Cancer
Source: Cancers (Basel). 2024 Jun 6;16(11):2154. doi: 10.3390/cancers16112154 (PMC11171784; doi:10.3390/cancers16112154)
Supplement: Supplementary file 1 [file cancers-16-02154-s001.zip › Supplementary Table S4.pdf]

Supplementary Table S4. Primer/probe sequences and amplicon size of pyrosequencing assays in this study.

Locus	Primer sequence
SORT1A	Fwd: 5’-BIO-GGGTTGATTTTAGTAGTTTTTG-3’
	Rev: 5’-CCCTACCCACTCCTCATTAA-3’
	Seq: 5’-CCTACCCACTCCTCATTA-3’
	PCR product: 114 bp
SORT1B	Fwd: 5’-GTGTTTGTTAGAGGTTTTAGGG-3’
	Rev: 5’-BIO-TAAAAAACTAACCTTCAAACTTCC-3’
	Seq: 5’-GTTAGAGGTTTTAGGGAG-3’
	PCR product: 103 bp
LINE-1	Fwd: 5’-BIO-TAGGGAGTGTTAGATAGTGG-3’
	Rev: 5’-AACTCCCTAACCCCTTAC-3’
	Seq: 5’-CAAATAAAACAATACCTC 3’
	PCR product: 108 bp

Thermal profiles were as follows: 95°C for 15 min followed by 40 cycles of 94°C for 30 sec, 55°C for 30 sec (SORT1A and SORT1B) or 58°C for 45 sec (LINE-1) and 72°C for 45 sec and a final extension step at 72°C for 10 min.
