# Supplementary figures and images for: Novel Transcriptional and DNA Methylation Abnormalities of SORT1 Gene in Non-Small Cell Lung Cancer
Source: Cancers (Basel). 2024 Jun 6;16(11):2154. doi: 10.3390/cancers16112154 (PMC11171784; doi:10.3390/cancers16112154)

Supplementary figure S1

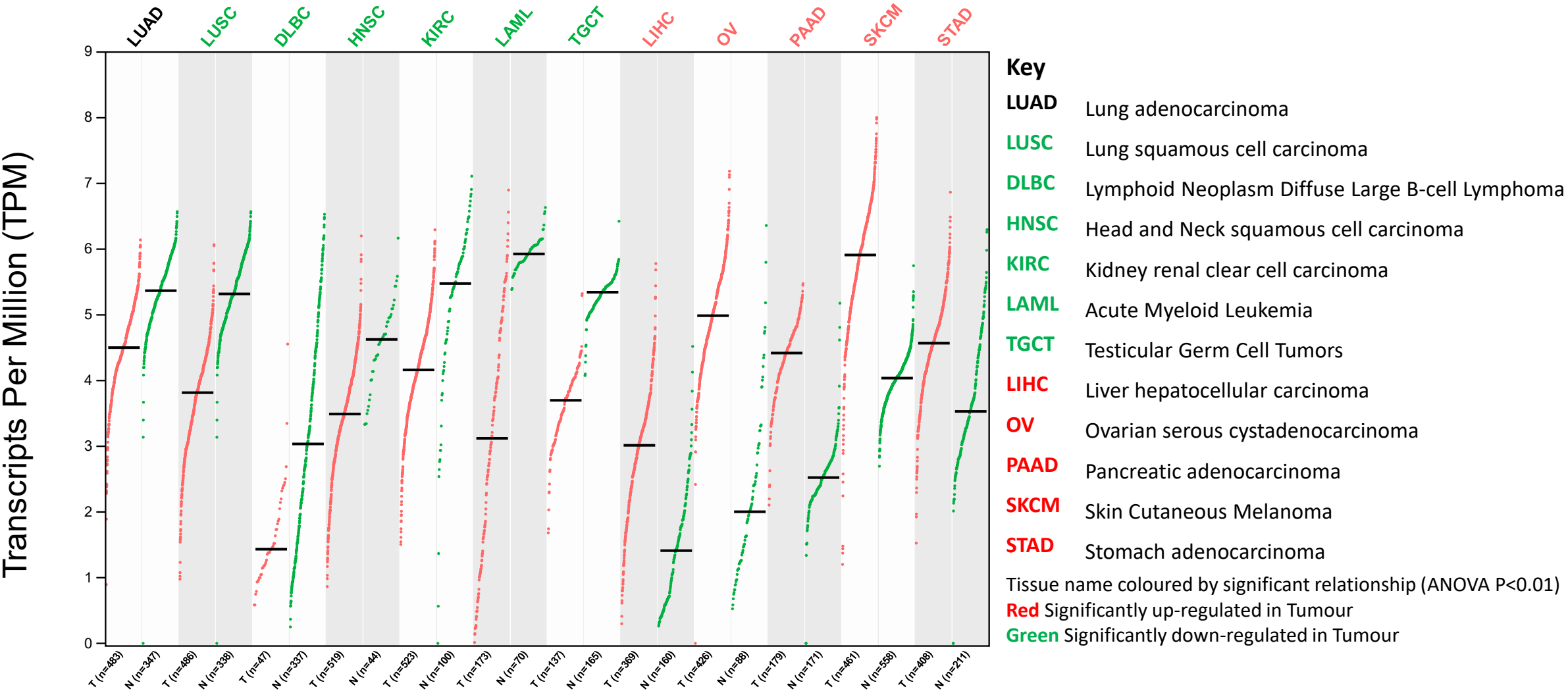

Supplement: Supplementary file 1 [file cancers-16-02154-s001.zip › Supplementary Figure S1.pdf]

Supplementary figure S2

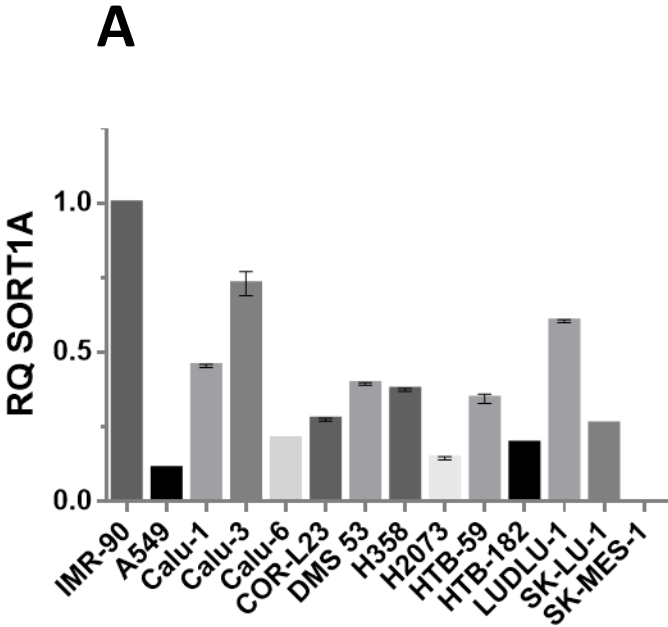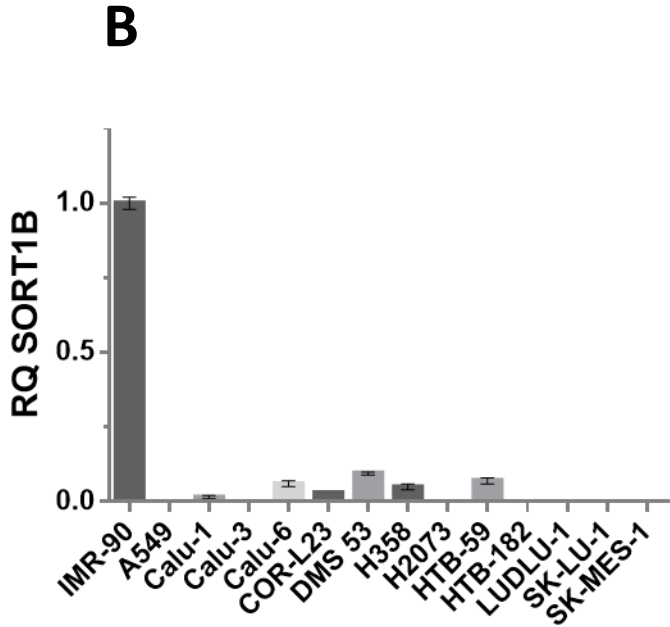

Supplement: Supplementary file 1 [file cancers-16-02154-s001.zip › Supplementary Figure S2.pdf]

## SORT1A1

## SORT1A2

<http://swissmodel.expasy.org/>

**A**

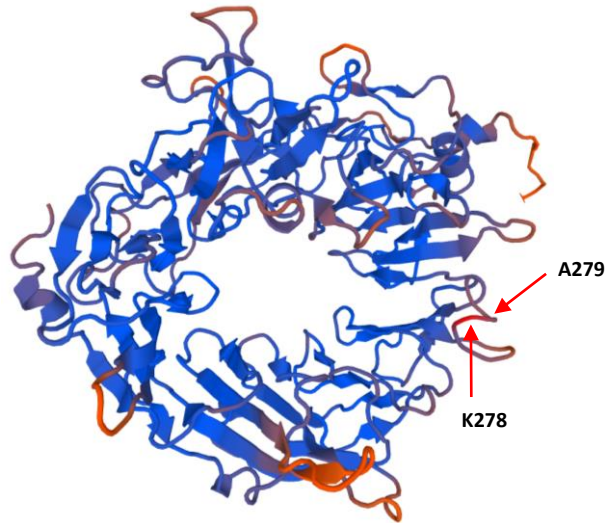

**B**

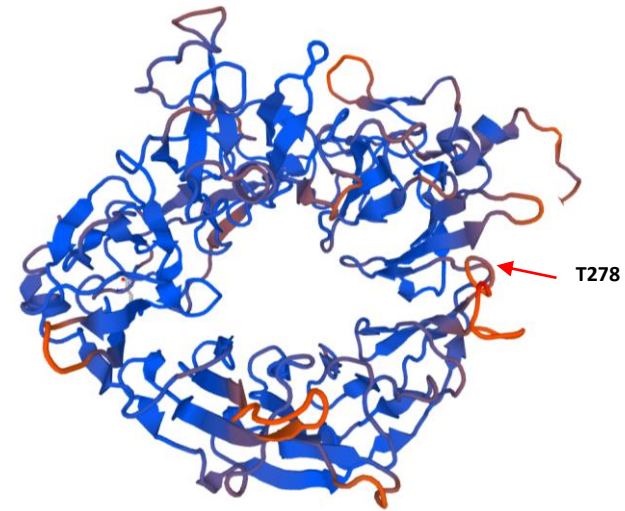

**C**

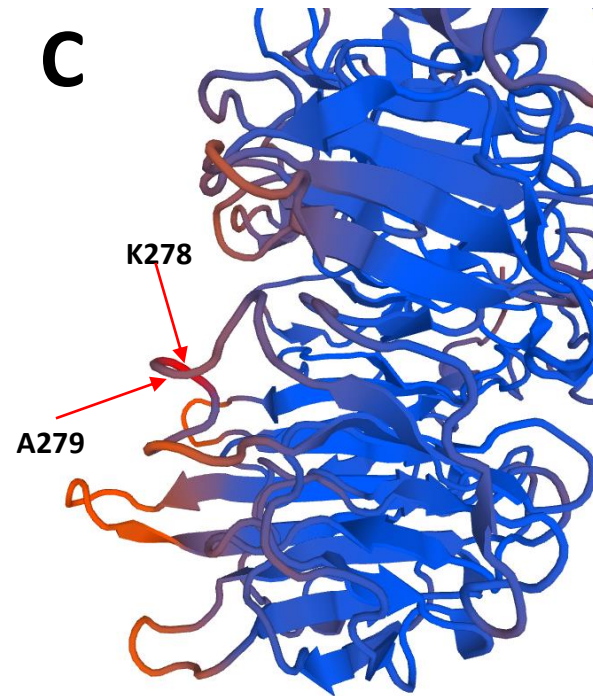

**D**

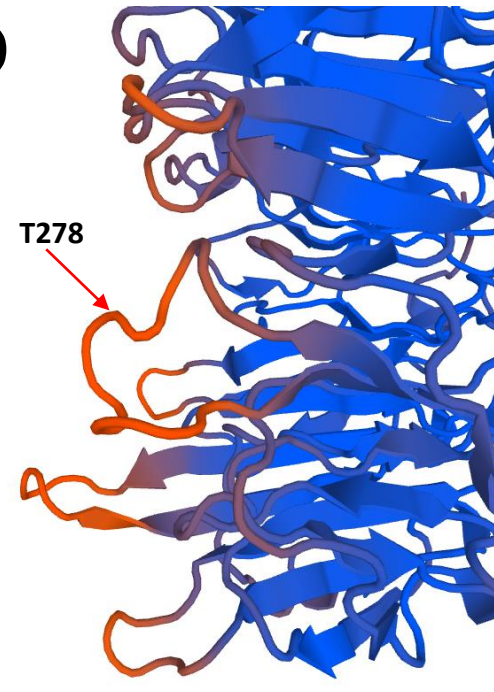

Supplement: Supplementary file 1 [file cancers-16-02154-s001.zip › Supplementary Figure S5.pdf]
